# Impacts of Forest Bathing (Shinrin-Yoku) in Female Participants with Depression/Depressive Tendencies

**DOI:** 10.3390/diseases13040100

**Published:** 2025-03-28

**Authors:** Qing Li, Norimasa Takayama, Masao Katsumata, Hiroshi Takayama, Yukako Kimura, Shigeyoshi Kumeda, Takashi Miura, Tetsuya Ichimiya, Ruei Tan, Haruka Shimomura, Amane Tateno, Tsunemi Kitagawa, Yoichiro Aoyagi, Michiko Imai

**Affiliations:** 1 Department of Rehabilitation Medicine, Graduate School of Medicine, Nippon Medical School, 1-1-5 Sendagi, Bunkyo-ku, Tokyo 113-8603, Japan; t-kita@nms.ac.jp (T.K.); y.aoyagi@nms.ac.jp (Y.A.); 2 Forestry and Forest Products Research Institute, Forest Research and Management Organization, Tsukuba 300-1244, Japan; hanri@ffpri.affrc.go.jp; 3 Nursing School, Nippon Medical School, Chiba 270-1613, Japan; masao-k@nms.ac.jp; 4 Takayama Orthopedic Clinic, Tokyo 617-0814, Japan; tibiaspine@ymail.ne.jp; 5 itswellness org, Tokyo 305-8687, Japan; y_kimura22@itswellness.org; 6 Nagano Prefectural Kiso Hospital, Nagano 397-0001, Japan; kiso@pref-nagano-hosp.jp; 7 Agematsu Town Office, Nagano 399-5601, Japan; syoukan@town.agematsu.nagano.jp; 8 Ichimiya Mental Clinic, Tokyo 110-0005, Japan; ichimiya@i-mc.jp; 9 Tan Clinic, Tokyo 214-0001, Japan; tliuying1207@tan-clinic.com; 10 Hojo Clinic Mizonokuchi, Kawasaki 213-0011, Japan; simomura@sounkai.com; 11 Department of Neuropsychiatry, Graduate School of Medicine, Nippon Medical School, 1-1-5 Sendagi, Bunkyo-ku, Tokyo 113-8603, Japan; amtateno@nms.ac.jp; 12 INFOM (International Society of Nature and Forest Medicine), Tokyo, Japan; info@infom.org

**Keywords:** depression, female participants, forest bathing, insulin-like growth factor I (IGF-1), sleep, oxytocin, POMS, SDS, serotonin, shinrin-yoku

## Abstract

Background: It has been reported that forest bathing significantly reduced negative emotions and increased the positive feelings in both healthy males and females, as well as increasing blood serotonin in healthy males, indicating the potential for a beneficial effect on depressive status. However, an improvement effect of forest bathing on participants with depression has not been reported so far. Therefore, in order to fill this gap, this study examined the effect of forest bathing on depression in female participants with depression/depressive tendencies. Methods: Thirty-one females aged 40.1 ± 2.4 years with depression/depressive tendencies were recruited after obtaining informed consent. The study employed a randomized crossover design to compare forest bathing with city walking. They participated in day trips to a Japanese cypress forest park and to a city area of Nagano Prefecture as a control in June 2023. On both trips, they walked 2.5 km (for 90 min) in the morning and afternoon, respectively, for a total of 5.0 km per day. Blood samples were taken at 4 pm for the measurements before forest bathing on the first day and after the walking in forest and unban sites on the second and third days, at the same hospital. Concentrations of oxytocin, IGF-1, serotonin and lactic acid in blood were measured. SDS scores were calculated and the POMS test and questionnaires for subjective fatigue symptoms and sleep quality were administered before and after each trip. Temperature, humidity and illuminance were also measured in the forest and urban environments. The Nippon Medical School Central Ethics Committee approved this study. Results: Forest bathing significantly decreased SDS scores compared to city walk and the baseline, and the effect lasted for one week after forest bathing. Forest bathing also significantly increased the concentrations of blood serotonin in participants who were not taking antidepressants, significantly increased the levels of oxytocin and IGF-1 in blood, significantly increased the scores for positive feelings, and reduced the scores for negative emotions compared with city walking in the POMS test. In addition, forest bathing reduced subjective fatigue symptoms and improved sleep quality. Conclusions: These findings provided scientific evidence to contribute to understanding forest bathing as a potential intervention for preventing depression, and future research on males should further explore these effects.

## 1. Introduction

Researchers in Japan have tried to find preventive effects in forests against lifestyle-related diseases, and have proposed a new concept called “shinrin-yoku”, or “forest bathing” in English [1]. Forest bathing is an activity that utilizes the healing effects of forests to promote health and prevent disease through forest walks. Forest bathing exerts its effects by stimulating the five senses (sight, smell, hearing, touch and taste) [1]. Since 2004 many scientific studies on the psychological and physiological effects of forest bathing/shinrin-yoku have been conducted and many original scientific articles have been published [1,2]. It has been reported that forest bathing/shinrin-yoku boosted immune system by increasing human natural killer activity and anticancer proteins in natural killer cells [1,2,3,4], lowered blood pressure and pulse rate, showing preventive effects on hypertension and heart diseases [5,6], reduced stress hormones including adrenaline and noradrenaline in urine and cortisol in serum and saliva [3,4,5,6,7], increased parasympathetic nervous activity and decreased sympathetic nervous activity [5,6,7,8,9,10,11,12,13]. In addition, it has been reported that this practice improved sleep, showing preventive effect on sleep disorders [2,14], and reduced negative emotions and increased positive feelings, showing a potential preventive effect on depressive status [2,4,5,6,7,8,9,10,11,12,13,14]. Based on the above evidence, forest medicine as a new preventive medicine has been proposed [1,2]. On the other hand, depression is a common mental disorder worldwide, with an estimated 5% of adults suffering from depression (4% of men and 6% of women). Approximately 280 million people in the world have depression and more women are affected by depression than men [15]. In addition, worldwide, more than 10% of pregnant women and women who have just given birth experience depression [16]. This is a big social problem in the world. However, effective preventive measures against depression have not been established. Because forest bathing can reduce stress hormones, stabilize the autonomic nervous system, reduce negative emotions, increase positive feelings and has shown a relaxation effect in both males and/or females [3,4,5,6,7,8,9,10,11,12,13], forest bathing may be used in interventions aiming to prevent depression. It has been reported that patients with depression have lower blood serotonin levels [17,18,19,20,21,22]. Recently, we found that forest bathing significantly increased the levels of serotonin in serum and significantly improved feelings of sleepiness on rising and feeling refreshed in healthy males [14]. These findings suggested that forest bathing has the potential to improve depressive status. However, there has yet to be any research on the impact of forest bathing on blood serotonin levels in patients with depression/depressive tendencies.

Oxytocin plays a central role in human social behavior, social cognition, anxiety, mood, stress modulation, and fear learning and extinction. The relationships between oxytocin and psychiatric disorders, including depression, anxiety and autism spectrum disorder, have been extensively studied [23,24,25,26]. These findings indicate an association between depressive symptomatology and oxytocin levels. In addition, oxytocin is of particular interest because it has been shown to reduce anxiety through down-regulation of the hypothalamic–pituitary–adrenal (HPA) axis [24,26,27]. Forest bathing also affects the HPA axis by reducing adrenaline and cortisol [2,3,4,5,6]. It has been reported that insulin-like growth factor I (IGF-1) increases the number of new neurons in the hippocampus, contributing to antidepressant effects [28,29,30,31]. Pregnant women with a high serum IGF-1 concentration in the first trimester were less likely to develop postpartum depression than those with a low concentration. A high serum IGF-1 concentration during pregnancy may help to protect against postpartum depression development [32]. Taş Dürmüş et al. reported that a high-intensity interval training program resulted in significant reduction in the symptoms of depression, anxiety as evaluated by the Hamilton Depression Rating Scale and the Hamilton Anxiety Rating Scale, and increased the serum IGF-1 levels [33] suggesting a strong relationship between depression and IGF-1. However, there has yet to be any research on the impacts of forest bathing on blood oxytocin and IGF-1 levels in patients with depression.

The Self Rating Depression Scale (SDS) test is a tool developed in 1965 for assessing emotional disturbance, including depression [34]. However, there have been no studies on the impact of forest bathing on SDS in participants with depression/depressive tendencies so far. The novelty of this study is to examine the impacts of forest bathing in depressed participants.

Against this background, we hypothesized that forest bathing may have beneficial impacts for patients with depression. Differing rates of depression between the sexes have been reported in the United States and elsewhere, and females show higher rates than males [35,36,37,38]. Brody and Hughes reported that, overall, women (10.4%) were almost twice as likely to experience depression as men (5.5%) in the United States from 2013–2016. This pattern was also observed in other age groups [38]. In addition, we previously found that, although the detailed mechanism is unknown, the effect of forest bathing was greater in women than in men in terms of subjective symptoms and stress hormones [2,3,4]. Thus, the impacts of forest bathing on females with depression/depressive tendencies were explored in the present study.

In addition, we have clarified the preventive effects of forest bathing on hypertension [6] and on enhancing immune function [3,4] so far, but the preventive effect of forest bathing on depression is also very important. Therefore, taking the above background into account, we decided to study the preventive effect of forest bathing on depression as evaluated by SDS and on blood serotonin, oxytocin and IGF-1 in this study.

## 2. Methods

### 2.1. Participants

The Self Rating Depression Scale (SDS) test is a tool developed in 1965 for assessing emotional disturbance, including depression [34]. Since the SDS is simple, it can be used for assessment in a variety of patients. It is now covered by Japanese medical insurance and therefore has become a useful tool for assessing emotional disturbance in daily clinical practice in Japan. As shown in Table 1, the SDS has 20 items, and each item is scored from 1 to 4 points. The total score ranges from 20 to 80, and a higher score indicates depressive symptoms are serious [34,39]. Patients with SDS scores less than 40 are categorized as normal, those with scores between 40 and 49 as borderline, those with scores between 50 and 59 are considered depressive, and those with scores more than 59 as severely depressive [40].

In the present study, thirty-one female participants with SDS scores between 40 and 59 and ranging in age from 24 to 66 years (mean ± standard error: 40.1 ± 2.4 years) were recruited from three clinics (Ichimiya Mental Clinic in Tokyo, Tan Clinic in Tokyo and Hojo Clinic Mizonokuchi in Kawasaki, Japan). Information gathered from a self-administered questionnaire, including age and lifestyle habits, that asked about cigarette smoking, alcohol consumption, sleeping hours and physical exercise, have been reported previously [3,4]. Thirty-one participants were randomly divided into groups A and B (Table 2). Half of the subjects from each clinic were randomly assigned to Group A and the other half to Group B. There was no significant difference between groups A and B in age, SDS score, depression-related medications, lifestyle habits including cigarette smoking, alcohol consumption, sleeping hours and physical exercise, white blood cell counts (WBC), red blood cell counts (RBC), hemoglobin, hematocrit or platelet counts. Written informed consent was obtained from all participants after a full explanation of the study procedures. The informed consent document provided details on how participants were fully informed about the study procedures, particularly regarding the potential risks or discomfort associated with participation. All participants stayed in the same hotel and ate the same meals. To eliminate the impact of alcohol, the participants abstained from alcohol during the experiment. This experiment was carried out in accordance with the Declaration of Helsinki, and was approved by Nippon Medical School Central Ethics Committee.

### 2.2. Forest Bathing and Urban Walks

**Figure 1:**
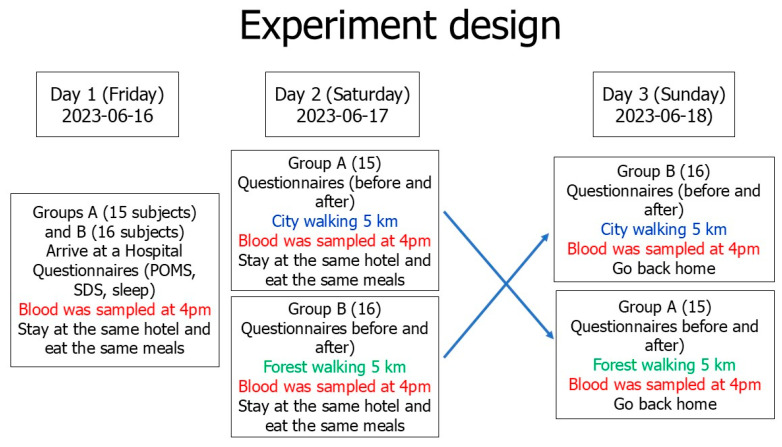
A randomized crossover research design and methods used in the experiment.

**Figure 2:**
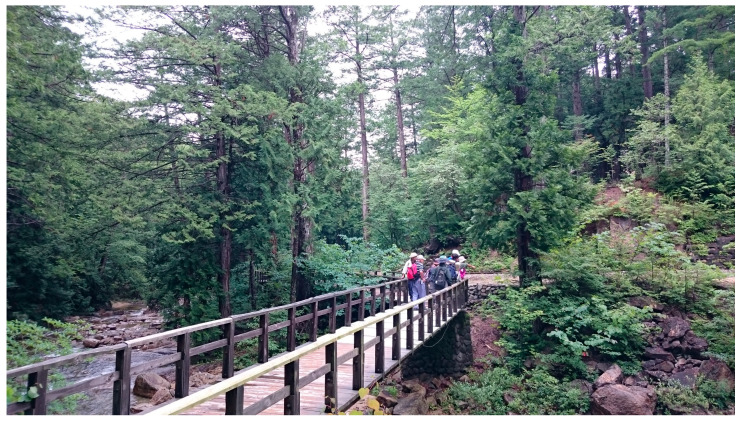
Forest walking route and walking scene (males in Figure 2 are staff, not participants).

**Figure 3:**
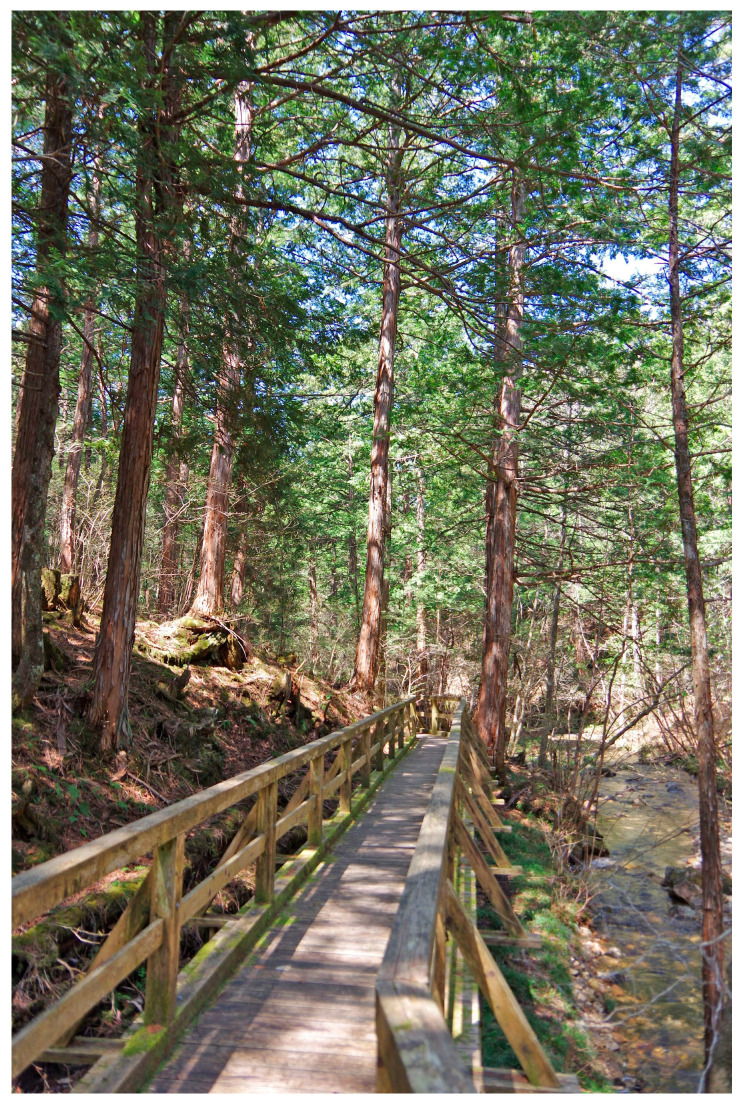
Wheelchair-accessible forest walking route.

A randomized crossover design was used, as shown in Figure 1 and as reported previously [14]. The participants participated in a three-day trip to a Japanese cypress forest park in Nagano Prefecture and to city areas with almost no trees in June 2023 as a control. Most of the trees in the Japanese cypress forest are conifers, with very few broad-leaved trees (Figure 2 and Figure 3). 

Because a crossover design is a valuable and powerful research method, especially for comparing interventions within the same subjects, we employed a randomized crossover design to compare forest bathing with city walking in the present study. The advantages of the crossover design are as follows:

1. Increased efficiency: Each participant serves as their own control, which minimizes the variability caused by differences between individuals. This requires fewer participants to achieve statistically significant results.
2. Direct comparisons: Participants receive the interventions (forest bathing and city walking) in a randomized order, making it easier to compare the effects of different interventions directly.
3. Control of individual differences: Since each participant experiences both interventions (forest bathing and city walking), the design controls for individual differences in response to interventions, leading to more reliable results.
4. Cost-effectiveness: With fewer participants needed, the overall costs of the study can be reduced, making it more feasible to conduct.
5. Because we used the randomized crossover design, all subjects experienced forest bathing and urban walking, even though the participants experienced the forest and city treatments on different dates. The differences in participants’ responses to the two different environments could be analyzed.

On the other hand, the crossover design has the following limitations:

1. Carryover effects: There is a risk that the effects of one treatment may carry over into the next period, potentially confounding results.
2. Not suitable for all conditions: Crossover designs may not be appropriate for treatments that have long-lasting effects or when the condition being studied cannot be reversed.
3. Dropout issues: If participants drop out after receiving one treatment, it can lead to imbalances in the groups and affect the validity of the results.

However, there is no perfect study design. This study adopted a randomized crossover design because the advantages of the crossover design outweighed the limitations.

As shown in Figure 1, on the first day, all participants departed from Tokyo and arrived at a hospital near the forest park, where their blood was sampled at 4 pm as the baseline. The SDS scores were evaluated and the POMS test and questionnaires for subjective fatigue symptoms and subjective sleep quality were conducted before blood collection as baseline controls of forest bathing and city walking. All participants stayed in the same hotel and ate the same meals. To minimize potential biases in the baseline assessments, participants were asked to abstain from alcohol for two days before the start of the experiment, and after gathering on the morning of the first day, participants performed the same activities and ate the same lunch.

On the second day, group A was randomly assigned to the city site and group B was assigned to the forest site, as shown in Figure 1. On the third day, the participants switched field sites. Information about altitude (height above sea level) and walking time, distance, and speed in the forest park and city area are shown in Table 3.

**Figure 4:**
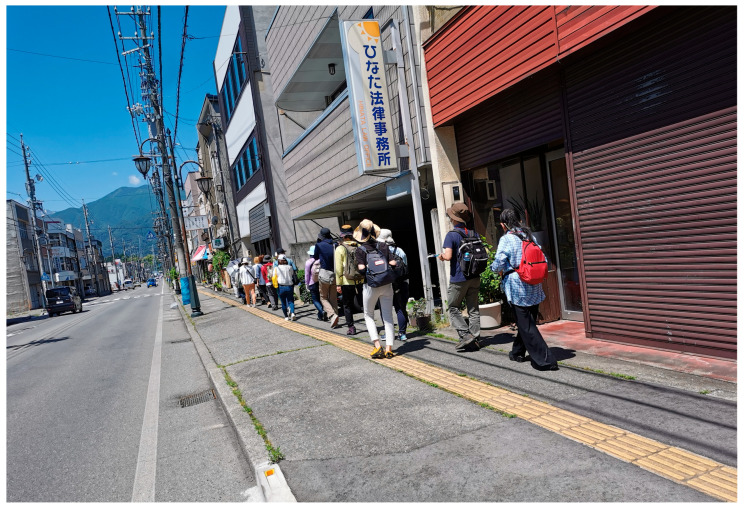
City walking route and walking scene (males in Figure 4 are staff, not participants). The non-English term in the figure is a company sign.

Both the forest walking route (Figure 2 and Figure 3) and the city walking route (Figure 4) are flat roads, and the forest walking route is wheelchair accessible (Figure 3). Therefore, the impact of the differences in the inclines and declines between city walks and forest walks should be limited.

Temperature, humidity and illuminance were also measured in the forest and urban environments [14]. It was sunny on both days in the city and in the forest. As shown in Table 4, illuminance and temperature in the city environment were significantly higher than in the forest environment, whereas the humidity in the forest environment was significantly higher than in the city environment. Although we did not measure wind speed, we collected data from local meteorological observatories. The wind speeds on 17 June around the forest and city were 1.7 m/s and 1.4 m/s and the difference was 0.3 m/s; on 18 June, around the forest it was 0.61 m/s, and in the city, it was 1.1 m/s, and the difference was 0.5 m/s. Therefore, the wind power in both the forest and the city on both days was classified as light air/light breeze. The difference in wind speed between city walks and forest walks was quite limited.

### 2.3. Blood Tests and Questionnaire Surveys

#### 2.3.1. Serotonin in Serum

The concentration of serotonin in serum was measured by Bio Medical Laboratories (BML), Inc. in Tokyo, Japan with the high performance liquid chromatography (HPLC) method, as described previously [14]. The normal range of serotonin levels in serum is 81.0–262.0 ng/mL. The detection rate of serotonin was 100% in the present study.

#### 2.3.2. Oxytocin in Plasma

The relationships between oxytocin and psychiatric disorders including depression, anxiety, and autism spectrum disorder have been extensively studied [23,24,25,26,27]. Thus, in the present study, blood oxytocin was measured to examine the effect of forest bathing on oxytocin in depressed participants. The concentration of oxytocin in plasma was measured by enzyme immunoassay with an Oxytocin ELISA kit (Enzo Life Sciences, Farmingdale, NY 11735, USA) [41]. The detection limit is 2.5 pg/mL and the detection rate of oxytocin was 100% in the present study.

#### 2.3.3. Insulin-like Growth Factor 1 (IGF-1) Concentration in Plasma

It has been reported that IGF-1 increases the number of new neurons in the hippocampus, contributing to antidepressant effects [28,29,30,31,32,33]. Thus, in the present study, blood IGF-1 was measured to examine the effect of forest bathing on IGF-1 in depressed participants. The concentration of IGF-1 in plasma was measured by BML with an electro chemiluminescence immunoassay (ECLIA). The normal range of IGF-1 levels in plasma is 59–245 ng/mL. The detection rate of IGF-1 was 100% in the present study.

#### 2.3.4. Lactic Acid Concentration in Serum

Because exercise increases blood lactate, blood lactate concentration is an index that objectively evaluates the amount of exercise and physical activity. This study measured blood lactate concentrations to monitor the differences in exercise volume between urban walking and forest bathing. Blood lactic acid concentration was measured as described previously [14]. The detection rate of lactic acid was 100% in the present study.

#### 2.3.5. SDS Scores

The SDS is a simple questionnaire to evaluate depression. Participants with SDS scores less than 40 are categorized as normal, those with scores between 40 and 49 as borderline, those with scores between 50 and 59 are considered depressive, and those with scores more than 59 as severely depressive [40]. SDS scores were evaluated using the Japanese version of the SDS evaluation sheet, as described in the section on participants and in Table 1.

#### 2.3.6. POMS Test

The POMS test is a simple questionnaire to evaluate mental stress and depression [14]. As shown in Table 5, POMS 2 questionnaire in Japanese is a short version, consisting of 35 questions.

Before and after the trips, the POMS test was conducted, using the POMS 2 questionnaire in Japanese for adults, as described previously [14].

#### 2.3.7. Questionnaire for Subjective Fatigue Symptoms

As shown in Table 6, the questionnaire for subjective fatigue symptoms consists of 30 items and is divided into Groups I, II, and III. Group I (10 items) evaluates sleepiness and sluggishness (decreased vitality), group II (10 items) evaluates difficulty in concentrating attention (decreased energy), and group III (10 items) evaluates fatigue symptoms such as physical discomfort. Groups I and III can be said to be physical symptoms, and Group II can be said to be psychological symptoms. The questionnaire has previously been applied to evaluate the impacts of forest bathing [2]. This questionnaire was conducted before and after the trips.

#### 2.3.8. Questionnaire for Subjective Sleep Quality

As shown in Table 7, the questionnaire of the Oguri-Shirakawa-Azumi sleep inventory MA version was used. OSA-MA consists of 16 items and has a five-factor structure, including factor 1 (sleepiness on rising), factor 2 (initiation and maintenance of sleep), factor 3 (frequent dreaming), factor 4 (feeling refreshed on rising), and factor 5 (sleep length). Responses were scored on a 4-point scale ranging from 0 to 3, and all scores were converted into 5 subscale scores using the OSA-MA standardized scoring program [42,43,44]. The OSA-MA scores were calculated as corrected (Zc) scores, with higher scores indicating better quality of sleep [42]. This questionnaire was conducted before and after the trips.

Subjective measures to assess fatigue and sleep quality in the present study may be influenced by individual perceptions; however, the effect of forest bathing on sleep has been confirmed by objective measures such as polysomnography [45] and piezoelectric accelerometer measurements (Actiwatch^®^, Mini Mitter Co., Inc., Sunriver, OR, USA), which can monitor the sleep time and daily physical activity of the subjects [46].

### 2.4. Statistical Analysis

A paired *t*-test was used to compare the differences between forest and city walks and between before and after the forest and city walks in lactic acid concentrations, levels of serotonin, oxytocin and IGF-1 in blood, and scores in the POMS and SDS tests and questionnaires for subjective fatigue symptoms and subjective sleep quality, as described previously [14]. A *t*-test was used to compare the differences between the forest and city walks in environmental illuminance, temperature, and humidity.

## 3. Results

### 3.1. Walking Time, Distance, and Speed

As shown in Table 3, there were no significant differences in the walking time, distance, and speed of the guides who guided the participants between city and forest areas.

### 3.2. Lactic Acid Concentrations in Serum During the Forest Bathing and Urban Area Walking

As shown in Table 8, there were no significant differences in lactic acid concentrations between forest and city walks, before and after forest walk or before and after city walk (all *p* > 0.05).

### 3.3. Impacts of Forest and City Walks on Blood Serotonin

Twenty-two participants took antidepressants, while nine did not take antidepressants during the study, as shown in Table 9, Table 10 and Table 11.

As shown in Table 9 and Table 10, the serotonin levels in serum of the participants varied greatly, depending on whether they were taking antidepressants. Because of this, statistical analyses were conducted separately for participants taking antidepressants and those not taking them.

Table 9 shows the results of serotonin in serum in participants who took medications. According to data from a serotonin measurement company (BML), the normal range of serotonin levels in serum is 81.0–262.0 ng/mL. However, the average serotonin concentrations in serum before walking in the participants who took medications was 30.25 ng/mL, which is significantly lower than 81.0 ng/mL, the lower limit of the normal range of serotonin in serum. In addition, there were 12 participants (12/22) with levels below 10.0 ng/mL.

As shown in Table 9, there were no significant differences in serotonin between the forest bathing and city walking treatments, before and after forest bathing, or before and after city walking in participants who took medications (all *p* > 0.05).

On the other hand, as shown in Table 10, in the participants who did not take medications, the difference between measurements after forest bathing and after city walking was significant (*p* = 0.042) (Table 10 and Table 11), suggesting that the forest walk significantly increased blood serotonin.

The serotonin concentrations in serum before walking in all participants who did not take medications were higher than 81.0 ng/mL (Mean: 136.79 ng/mL).

The average value for participants who took antidepressant medications (30.25 ng/mL) was significantly lower than that of the participants who did not take them (136.79 ng/mL) (*p* < 0.0001). This suggests that antidepressants significantly reduce blood serotonin concentration.

### 3.4. Impacts of Forest and City Walks on Blood Oxytocin

As shown in Table 12, blood oxytocin concentrations after a walk in the forest and after a walk in the city were both significantly higher than before (*p* = 0.0014, *p* = 0.011, respectively). The concentration after forest bathing was significantly higher than that after city walking (*p* = 0.031), suggesting that forest bathing increases the blood oxytocin concentration more than does city walking.

A significant negative correlation was observed between oxytocin and age (r = −0.351, n = 31, *p* < 0.05). In contrast, the concentrations of oxytocin were 8.80 ± 2.90 pg/mL (mean ± SD, n = 22) in participants who took antidepressants and 7.28 ± 1.57 pg/mL (mean ± SD, n = 9) in participants who did not take the antidepressants, with no significant difference between the two groups (*p* = 0.071), indicating that antidepressants did not affect the level of blood oxytocin.

### 3.5. Impacts of Forest and City Walks on Blood IGF-1

As shown in Table 13, the blood IGF-1 concentration after forest bathing was significantly higher than that after city walking (*p* = 0.016), suggesting that forest bathing increases the blood IGF-1 concentration.

### 3.6. Impacts of Forest and City Walks on SDS Scores

As shown in Table 14, there was no significant difference between SDS at the time of recruiting participants and SDS from the day before forest bathing (Friday 16 June). We thus took the SDS from the day before forest bathing as the baseline SDS and compared it with the SDS scores after forest bathing and after city walking. Both forest bathing and city walking significantly reduced SDS scores compared to the baseline (all *p* < 0.01). In addition, the SDS score after forest bathing was significantly lower than that after city walking (*p* < 0.01), indicating that forest bathing has a greater impact than city walking. Moreover, the SDS score after one week remained significantly lower than that before forest bathing, suggesting that this impact was sustained for one week after forest bathing.

### 3.7. Impacts of Forest and City Walks on the Scores in the POMS

The impacts of forest and city walks on POMS scores are shown in Table 15. Scores for AH (anger–hostility), CB (confusion–bewilderment), DD (depression–dejection), FI (fatigue–inertia), TA (tension–anxiety), VA (vigor–activity), F (friendliness) and TMD (total mood disturbance) were significantly improved after forest bathing compared with before forest bathing (all *p* < 0.01). Moreover, scores for AH, CB, FI, TA, VA, F and TMD were also significantly improved after forest bathing compared with after city walking (all *p* < 0.05). scores for VA (vigor–activity) and F (friendliness) were significantly higher after forest bathing than after city walking (*p* < 0.01), suggesting a beneficial effect of forest bathing in POMS scores. Scores for CB, TA and VA were also significantly improved after city walking compared with before (all *p* < 0.05).

### 3.8. Impacts of Forest Bathing on Subjective Fatigue Symptom Scores

Table 16 shows the impact of forest and city walks on subjective fatigue symptoms. Group I evaluates sleepiness and sluggishness (decreased vitality), group II evaluates difficulty in concentrating attention (decreased energy), and group III evaluates fatigue symptoms such as physical discomfort, as reported previously [2]. It was found that forest bathing significantly lowered the scores of Groups I, II and III compared to before, and significantly lowered the scores of Groups I and III compared with city walking, indicating that forest bathing was effective in improving subjective fatigue.

### 3.9. Impact of Forest and City Walks on Subjective Sleep Quality

As shown in Table 17, forest bathing significantly improved sleepiness on rising (*p* = 0.022) and sleep length (*p* = 0.036). Forest bathing also showed trends of improvement in feeling refreshed (recovery from fatigue), initiation and maintenance of sleep and frequent dreaming, but the differences were not significant, as assessed by the OSA-MA.

On the other hand, city walking did not show any positive impact on subjective sleep quality. In contrast, city walking significantly worsened frequent dreaming.

## 4. Discussion

This study was the first to comprehensively examine the impacts of forest bathing on female participants with depression and depressive tendencies, using indicators such as SDS, POMS, serotonin, oxytocin, IGF-1, subjective sleep quality, and subjective fatigue symptoms. We found that forest bathing improved SDS scores and that this impact lasted for one week after the forest bathing. Forest bathing also reduced negative emotions such as anger–hostility, confusion–bewilderment, depression–dejection, fatigue–inertia, tension–anxiety, total mood disturbance and subjective fatigue symptoms, while it increased positive feelings such as vigor–activity and friendliness in the POMS test, corroborating previous reports of such effects in healthy male and female participants [4,5,6,7,9,10,14]. In addition, forest bathing also reduced the scores of subjective fatigue symptoms.

Many studies have reported the relationship between depression and serotonin [14,17,18,19,20,21,22,47,48]. Against this background, we previously conducted an experiment and found that forest bathing/shinrin-yoku significantly increased blood serotonin in male participants without depression [14]. However, there had previously been no research on the impact of forest bathing/shinrin-yoku on serotonin in female patients with depression.

In this study, we found that patients with depression who took antidepressants had significantly lower concentrations of serotonin in blood serum compared with participants who did not take antidepressants. It has been reported that treatment with SSRI antidepressants such as sertraline [48], fluoxetine [49] and citalopram or escitalopram [50] reduces serotonin levels in plasma and/or in serum and that plasma serotonin levels are lower in depressive participants compared to controls. Baseline plasma serotonin levels did not correlate significantly with baseline HDRS scores in all depression participants [48].

In the present study, no increase in blood serotonin levels was observed, either after forest bathing or after taking a walk in the city, among participants who had taken antidepressants, and no impact of forest bathing was observed. The impact of antidepressants on blood serotonin levels far exceeded the impact of forest bathing, so it is thought that any impacts of forest bathing would have been masked by the antidepressants. Further research on this issue is required in the future.

In contrast, forest bathing/shinrin-yoku significantly enhanced blood serotonin in participants who did not take antidepressant medications compared to city walking, indicating a beneficial effect of forest bathing on blood serotonin in serum and reproducing our previous results in male participants [14].

Participants who took medication had significantly higher SDS scores (51.64) than those who did not take medication (46.44) (*p* < 0.05), suggesting that participants who took medications had stronger depressive symptoms.

As shown in Table 9 and Table 10, we examined the effects of forest bathing by dividing the participants into two groups, one taking antidepressants and the other not, which allowed us to control for the effects of antidepressants in the study design to accurately assess the impact of forest bathing on serotonin levels.

In addition, antidepressants significantly reduced blood serotonin levels, suggesting that the potential effect of forest bathing on serotonin was overshadowed by antidepressants in subjects who took antidepressants. Therefore, the effect of forest bathing should be evaluated for these participants using other indicators (oxytocin, IGF-1, SDS, etc.).

Oxytocin, a neuropeptide synthesized by the hypothalamus, plays a central role in human social behavior, social cognition, anxiety, mood, stress modulation, and fear learning and extinction. The relationships between oxytocin and psychiatric disorders including depression, anxiety, schizophrenia, and autism spectrum disorder have been extensively studied [23]. Ozsoy et al. [24] reported that female patients with depression had significantly lower oxytocin levels than control females, whereas no difference was found between male patients experiencing depression and male controls. Furthermore, antidepressant treatments appear to have no impact on serum oxytocin levels. Moreover, Veiga et al. (2022) [25] reported that depressive symptomatology was negatively associated with oxytocin serum levels in healthy female university students. These findings indicate an association between depressive symptomatology and oxytocin levels. In the present study we investigated for the first time the impact of forest bathing on oxytocin, and found that forest bathing significantly increased the level of oxytocin in plasma compared with city walking, indicating a beneficial effect of forest bathing on oxytocin in patients with depression. Improved friendliness (Table 15) in the POMS test after forest bathing supported this finding. It has been reported that social dialogue increased measured oxytocin in saliva [51]. Oxytocin can function as a stress-coping molecule, an anti-inflammatory and an antioxidant, with protective effects, especially in the face of adversity or trauma. Oxytocin influences the autonomic nervous system and the immune system. These properties of oxytocin may help explain the benefits of positive social experiences and have drawn attention to this molecule as a possible therapeutic in a host of disorders [52]. This is a new result among the impacts of forest bathing in patients with depression. In addition, antidepressant treatments appeared to have no impact on blood oxytocin levels, which is consistent with previous research [24].

It has been reported that IGF-1 increases the number of new neurons in the hippocampus, contributing to antidepressant effects and preventing cognitive decline [28,29,30,31,32,33]. It is known that exercise-induced increase in serotonin promotes the release of IGF-1 in the hippocampus, increases hippocampal neurogenesis through the IGF-1 signaling pathway, and produces antidepressant effects [30,33]. Thus, we investigated the impact of forest bathing on IGF-1 and found that forest bathing significantly increased blood IGF-1 compared with city walking, indicating a beneficial effect of forest bathing on IGF-1 in patients with depression. The finding that forest bathing increases the level of IGF-1 in patients with depression is new.

It is well known that sleep disorders, and sleep disturbance are common and key symptoms that affects most patients with depression [53,54]. We previously found that forest bathing improved subjective sleep quality in middle-aged males without depression, as evaluated by the OSA-MA questionnaire [14]. Thus, in the present study, the impacts of forest bathing on subjective sleep quality [42] in female participants with depression were also investigated, and we found that forest bathing significantly improved sleep quality and sleep length. It has been reported that forest bathing significantly increased sleep time [2]. Morita et al. [55] reported that forest walking improved sleep quality for individuals with sleep complaints. Kim et al. conducted a 6-day forest therapy program in postmenopausal women and measured their sleep situation by polysomnography and sleep questionnaires before and after the program. Polysomnography showed that sleep efficiency was increased. The total sleep time also increased, suggesting that forest therapy could be a good alternative to pharmacological treatment for mitigating insomnia in postmenopausal women [45]. Other studies also reported the benefits of forest bathing on sleep [56,57,58]. As shown in Table 16, we also found that forest walking improved subjective fatigue symptom scores, including sleepiness.

In this forest bathing experiment, the participants walked for two hours in the morning and two hours in the afternoon, including a lunch break, for a total of five hours in the forest per day. It has been reported that the longer the time spent in the forest and the more frequently forest bathing takes place, the greater the effects of forest bathing [1,2].

Physical activity affects mental health and depression biomarkers [59]. Therefore, we also controlled the impact of physical activity. In fact, participants walked the same distance during the same period in both trips, guided by forest therapists. The lactic acid concentration in serum is a useful indicator to evaluate physical activity [14]. There was no significant difference in blood lactic acid between participants who took part in the forest and city walks.

The altitude, which means height above sea level, differed between walking in the forest and walking in the city. Regarding the impact of the difference in the altitude between walking in the forest and walking in the city, multiple studies suggest that the risks of depression and suicide increase with increasing altitude of residence, and elevation appears to be a significant risk factor for depression [60,61]. Wang et al. (2019) [62] provide the first evidence that the prevalence of depression in Tibetans of the Qinghai-Tibet Plateau is higher than that in the general Chinese population and that reported in Western studies, a finding that may be related to cultural differences and chronic hypoxia caused by the high altitude. However, no studies were found regarding the impacts of short stays (one day) in high-altitude areas on depressive symptoms.

In this study, the altitude of forests (1110–1171 m) was higher than that of urban areas (631–647 m), therefore although high altitude may reduce the impact of forest bathing on improving depressive symptoms, it does not increase the impacts of forest bathing. In other words, the impacts of forest bathing on depression at high altitudes in the forest may be underestimated, but they are not overestimated. Therefore, the difference in altitude between forest and urban areas does not affect the conclusions of this study.

The city environmental illuminance was significantly higher than that in the forest areas. It has been reported that bright light therapy (≥1000 lx) is as an effective treatment for depression [63] indicating that although the impacts of forest bathing on depression at lower levels of environmental illuminance in the forest may be underestimated, they are not overestimated. Therefore, the difference in environmental illuminance between forest and urban areas does not affect the conclusions of this study.

On both days, the temperatures in the city were significantly higher than in the forest park, whereas the humidity in the forest park was significantly higher than in the city areas. It has been reported that weather conditions influence depressive symptoms [64]. On the other hand, the impacts of forest bathing are the total impact of the forest environment including the quiet atmosphere, beautiful scenery, calm climate, pleasant aromas, and clean fresh air compared with city environments which are affected by the weather conditions. In addition, beneficial (inhalable) biogenic volatile organic compounds that are present in forests but not in urban settings also contributed to this effect.

In fact, the effects of forest bathing are the total effects of the five senses of sight, hearing, taste, smell and touch, stimulated by the forest environment [1,2]. Therefore, the observed effects are influenced by audio-visual stimuli (senses of hearing and sight) and molecular components obtained from the trees, like volatile compounds (sense of smell). We previously found that volatile compounds from the trees (phytoncides) boosted the immune function, reduced stress hormones and improved emotions [65].

In addition to the mental health benefits, forest bathing has also been linked to improved immune function, which could contribute to viral disease prevention [2,3,65,66,67].

Although this forest bathing study was conducted in the Japanese forest environment, forest bathing is possible all over the world in similar forest environments. In fact, as a method of stress management, promoting health and/or preventing diseases, forest bathing/shinrin-yoku, which originated in Japan, is now spreading all over the world and becoming a focus of worldwide public attention [1,2,68]. Thus, we suggest that our study may be generalizable.

### Limitations

There were some limitations to the present study.

1. Participants were recruited from three different clinics in Tokyo and Kawasaki. The geographic location or specific characteristics of these clinics may affect the generalizability of the results to a broader population of women with depression in other regions or countries. However, this is a field study, and recruiting subjects was very difficult as needed to participate in a three-day, two-night trip. The representativeness of these three clinics is a study limitation.
2. Only female participants were investigated, and male participants with depression should also be investigated. We intend to conduct a study including male participants with depression next time.
3. As another limitation of this study, various factors such as deep breathing, differences between forest and urban in ambient illuminance, temperature, and humidity, the inclines and declines during walking and the conditions underfoot while walking, may affect blood serotonin and oxytocin concentrations and symptoms of depression; however, in this study, it was not possible to exclude the influence of some of these factors. In addition, this study was a field study, not a laboratory experiment; therefore, it is difficult to control all confounding factors. This should be addressed in the future research. However, both the city walking route (Figure 4) and the forest walking route (Figure 2 and Figure 3) are flat roads, and the forest walking route is wheelchair accessible. Therefore, the impact of the differences in the inclines and declines between city walks and forest walks should be limited. In fact, the effects of forest bathing are the total effect of the forest environment, including the quiet atmosphere, beautiful scenery, calm climate, pleasant aromas, and clean fresh air compared with city environments.
4. The number of participants was only 31 and more participants should be investigated. However, since the forest bathing study measures many indicators in a field study, the number of participants is limited in one experiment. In addition, all participants had to stay at the same hotel to control their diet; however, there is no bigger hotel in Akasawa Shizen Kyuyourin and the hotel has a limited capacity, with a maximum of 31 subjects. In addition, it is necessary to keep a distance between the subjects when walking in the city, and when there are many people, the traffic will be affected; therefore, permission from the city authorities could be obtained. In consideration of various factors, the number of participants was finally limited to 31. On the other hand, although the number of participants was only between 12 and 20 [3,4,14] in our previous studies, statistically significant differences were obtained.
5. In this study, we examined how much forest bathing and urban walking changed each indicator compared to the baseline measures. We took several measures to minimize potential biases in the baseline assessments as much as possible. However, it is difficult to eliminate all potential bias in the baseline assessment in a field survey. This is also one of the limitations of this study.

Taken together, despite the above limitations, this study has the following strengths.

1. It is the first study to find that forest bathing increased blood serotonin, oxytocin and IGF-1 in females with depression/depressive tendencies.
2. This study is also the first to find that forest bathing improves SDS scores in female participants with depression/depressive tendencies. The impact was sustained for one week after forest bathing.
3. We used a randomized crossover research design to eliminate order bias and improve the statistical efficiency [14]. Although the impacts of the first forest bathing may have an impact on the city walking next day, even in this situation, forest bathing was found to be more effective at improving depressive symptoms and other indicators than city walking; therefore, this design does not affect the conclusions of this study, as there may be an underestimation, but no overestimation, of the impacts of forest bathing.

The present article does not state that forest bathing cures depression, but rather suggests that forest bathing may have a potential contribution to improving depression, due to various factors in addition to the beneficial effects of forest bathing itself.

## 5. Conclusions

Our study indicated that forest bathing showed the following benefits in female participants with depression/depressive tendencies:

(1) Improvement in oxytocin, serotonin and IGF-1 in blood.
(2) Improvement in SDS, the effect of which lasted for one week.
(3) Improvement subjective sleep quality as assessed by the OSA-MA.
(4) Decrease in negative moods and increase in positive feelings of vigor and friendliness in the POMS test.
(5) Improvement in subjective fatigue symptoms.

These findings provided scientific evidence to contribute to understanding forest bathing as a potential intervention for preventing depression.

## 6. Directions for Future Research

(1) In the next phase of research, we plan to conduct a study on males with depression or participants with other mental illnesses.
(2) We would like to recommend exploring brain-derived neurotrophic factor and measurement of cerebral blood flow to strengthen the evidence for the therapeutic potential of forest bathing in future research.
(3) The present study was conducted in June, a time when both deciduous and evergreen trees emit phytoncides, which, when inhaled, could have a role in improving the studied parameters. We have previously conducted forest bathing experiments in this forest in autumn (September) [3], but have not conducted an experiment in winter. This will be the subject of future research. However, phytoncides in the air of this forest throughout the four seasons have been measured and the concentration of phytoncides was very low in winter [69].
(4) Future clinical research will verify the improvement of depression through forest bathing.

## Figures and Tables

**Table 1:** Description of the SDS questions and the scale used by participants to respond to them.

			Degree of	Condition	(Score)	
No.	Questions	No	Sometimes	Quite Often	Almost Always	Scores
1	Depressed affect	1	2	3	4	
2	Diurnal variation	1	2	3	4	
3	Crying spells	1	2	3	4	
4	Sleep disturbance	1	2	3	4	
5	Decreased appetite	1	2	3	4	
6	Decreased libido	1	2	3	4	
7	Weight loss	1	2	3	4	
8	Constipation	1	2	3	4	
9	Tachycardia	1	2	3	4	
10	Fatigue	1	2	3	4	
11	Confusion	1	2	3	4	
12	Psychomotor retardation	1	2	3	4	
13	Agitation	1	2	3	4	
14	Hopelessness	1	2	3	4	
15	Irritability	1	2	3	4	
16	Indecisiveness	1	2	3	4	
17	Personal devaluation	1	2	3	4	
18	Emptiness	1	2	3	4	
19	Suicidal ideation	1	2	3	4	
20	Dissatisfaction	1	2	3	4	
Total						

**Table 2:** Basic information on the participants in groups A and B.

	Group A (*n* = 15)		Group B (*n* = 16)		*p* Level
	Mean	SD	Mean	SD	
Age (years)	40.7	14.0	39.6	12.5	>0.05
SDS (Recruit) *	50.3	6.7	50.3	5.7	>0.05
Depression-related medications #	11/15		11/16		>0.05
Smoking #	2/15		2/16		>0.05
Alcohol #	4/15		5/16		>0.05
Daily exercise habits #	7/15		9/16		>0.05
Sleep time (h)	6.6	1.1	6.8	1.1	>0.05
WBC (×10^3^/μL)	5.9	1.4	6.3	1.9	>0.05
RBC (×10^6^/μL)	4.3	0.3	4.3	0.3	>0.05
Hemoglobin (g/dL)	12.4	1.0	12.8	0.9	>0.05
Hematocrit (%)	37.8	2.9	38.8	2.3	>0.05
Platelet (×10^3^/μL)	264	83.5	231.4	52.0	>0.05

*: “Recruit” means the SDS at the time of recruiting participants. #: number of “Yes”.

**Table 3:** Altitude and walking time, distance, and speed of guides for forest and city walks.

		Forest Area		City Area	
		a.m.	p.m.	a.m.	p.m.
17 June (Day 1)	Time	10:01–11:26	12:45–14:09	09:59–11:25	12:44–14:13
	Walking time	1:25:16	1:24:44	1:26:01	1:29:36
	Distance (km)	2.2	2.3	2.4	2.3
	Speed (km/h)	1.6	1.6	1.7	1.6
	Altitude (m)	1110–1158	1101–1166	633–646	632–645
18 June (Day 2)	Time	10:00–11:28	12:45–14:12	10:01–11:26	12:45–14:12
	Walking time	1:28:52	1:27:03	1:25:09	1:27:47
	Distance (km)	2.5	2.3	2.3	2.3
	Speed (km/h)	1.7	1.6	1.6	1.6
	Altitude (m)	1110–1165	1112–1171	634–647	631–645

**Table 4:** Environmental illuminance, temperature, and humidity in the forest and city areas during walks.

			Illuminance (lx)		Temperature (°C)		Humidity (%)	
Sites	Group	N	Mean	SD	Mean	SD	Mean	SD
Forest on 17 June	B	360	16,173.2 **	28,937.2	23.8 **	2.9	49.7 **	10.3
City on 17 June	A	338	56,398.4	49,372.2	34.2	4.3	23.4	8.4
Forest on 18 June	A	386	12,096.1 **	20,389.8	24.1 **	3.6	58.8 **	14.8
City on 18 June	B	335	58,500.1	33,539.2	34.9	4.2	26.6	7.1

**: *p* < 0.01 forest vs. city by *t*-test.

**Table 5:** Description of the POMS 2 questions and the scale used by participants to respond to them.

No.	Questions	Not at All	A Little	A Fair Amount	Quite a Lot	Very Much
1	I enjoy socializing	0	1	2	3	4
2	I feel tense	0	1	2	3	4
3	I feel angry	0	1	2	3	4
4	I feel exhausted	0	1	2	3	4
5	I feel lively	0	1	2	3	4
6	I feel confused	0	1	2	3	4
7	I care about others	0	1	2	3	4
8	I feel sad	0	1	2	3	4
9	I feel positive	0	1	2	3	4
10	I feel depressed	0	1	2	3	4
11	I feel full of energy	0	1	2	3	4
12	I feel confused	0	1	2	3	4
13	I feel hopeless	0	1	2	3	4
14	I feel anxious	0	1	2	3	4
15	I can’t concentrate	0	1	2	3	4
16	I’m tired	0	1	2	3	4
17	I feel like I can be useful to others	0	1	2	3	4
18	I feel nervous	0	1	2	3	4
19	I feel miserable	0	1	2	3	4
20	I can’t think clearly	0	1	2	3	4
21	I’m exhausted	0	1	2	3	4
22	I feel really angry inside	0	1	2	3	4
23	I worry about things	0	1	2	3	4
24	I can be kind to others	0	1	2	3	4
25	I can’t do anything myself	0	1	2	3	4
26	I feel fed up	0	1	2	3	4
27	I feel helpless	0	1	2	3	4
28	I feel very angry	0	1	2	3	4
29	I trust others	0	1	2	3	4
30	I get angry easily	0	1	2	3	4
31	I feel worthless	0	1	2	3	4
32	I feel energized	0	1	2	3	4
33	I’m not sure about things	0	1	2	3	4
34	I’m exhausted	0	1	2	3	4
35	I’m full of motivation	0	1	2	3	4

**Table 6:** Subjective fatigue symptoms questionnaire.

Name of participant:					Date:				
I would like to ask you about your current situation. If you have any of the following symptoms, please enter 1. If not, please enter 0.									
Group I			Group II				Group III		
No.	Symptoms	Score	No.	Symptoms		Score	No.	Symptoms	Score
1	My head is heavy	1 or 0	11	I can’t think straight		1 or 0	21	I have a headache	1 or 0
2	My whole body feels tired	1 or 0	12	I don’t like talking		1 or 0	22	My shoulders are stiff	1 or 0
3	My legs feel tired	1 or 0	13	I am frustrated		1 or 0	23	I have lower back pain	1 or 0
4	I yawn	1 or 0	14	Make more mistakes in doing		1 or 0	24	It’s hard to breathe	1 or 0
5	My brain is foggy	1 or 0	15	I am distracted		1 or 0	25	I am thirsty	1 or 0
6	Sleepy	1 or 0	16	I can’t be enthusiastic about things		1 or 0	26	My voice becomes hoarse	1 or 0
7	My eyes get tired	1 or 0	17	I can’t remember little things		1 or 0	27	I feel dizzy	1 or 0
8	My movement is clumsy	1 or 0	18	I care about things		1 or 0	28	My eyelids and muscles twitch	1 or 0
9	I can’t rely on my feet	1 or 0	19	I can’t stay tidy		1 or 0	29	My limbs tremble	1 or 0
10	I want to lie down	1 or 0	20	I’m running out of patience		1 or 0	30	I don’t feel well	1 or 0

**Table 7:** The sleep quality questionnaire using the Ogri-Shirakawa-Azumi sleep inventory MA version (OSA-MA).

No.	Question Items	Never	Seldom	Sometimes	Almost Always	Factor
1	I still felt tired	0	1	2	3	4
2	I couldn’t concentrate	0	1	2	3	1
3	I couldn’t sleep well	0	1	2	3	2
4	I felt stressed	0	1	2	3	1
5	I felt tired	0	1	2	3	4
6	I had no appetite	0	1	2	3	5
7	I dozed off a lot	0	1	2	3	2
8	I felt dazed	0	1	2	3	1
9	I had a lot of nightmares	0	1	2	3	3
10	I had trouble falling asleep	0	1	2	3	2
11	I felt uncomfortable	0	1	2	3	4
12	I had a lot of dreams	0	1	2	3	3
13	I woke up a lot	0	1	2	3	2
14	I was too embarrassed to answer	0	1	2	3	1
15	My sleep time was shorter	0	1	2	3	5
16	My sleep was shallow	0	1	2	3	2

**Table 8:** Impact of forest and city walks on lactic acid (mg/dL) (Mean ± SD).

	Before Walks (16 June)	After City Walking	After Forest Bathing
Mean	4.56 ± 1.91	4.73 ± 1.56	4.61 ± 1.31
N	31	31	31

**Table 9:** Serum serotonin concentration (ng/mL) in participants taking antidepressants (Mean ± SD).

	Age (Years)	Before (16 June)	After City Walking	After Forest Bathing	Scores of SDS
Mean	36.4 ± 11.3	30.25 ± 39.88	28.75 ± 38.38	28.50 ± 37.80	51.64 ± 6.15
N	22	22	22	22	22

**Table 10:** Serum serotonin concentration (ng/mL) of participants not taking antidepressants (Mean ± SD).

	Age (Years)	Before (16 June)	After City Walking	After Forest Bathing	Scores of SDS
Mean	49.2 ± 13.0	136.79 ± 38.77	132.60 ± 36.97	138.43 ± 40.59 *	46.44 ± 4.39
N	9	9	9	9	9

*: *p* < 0.05 (forest vs. city), paired *t*-test (two-tailed).

**Table 11:** Serum serotonin concentration (ng/mL) of participants not taking antidepressants within subject changes from baseline to after forest and city walking (Mean ± SD).

	Age (Years)	After City Walking	After Forest Bathing
Mean	49.2 ± 13.0	−4.19 ± 11.14	1.64 ± 8.48 *
N	9	9	9

*: *p* < 0.05: forest bathing vs. city walking by the paired *t*-test (two-tailed).

**Table 12:** Impacts of forest and city walks on the level of oxytocin in plasma (pg/mL) (Mean ± SD).

	Before Walk (16 June)	After City Walk	After Forest Bathing
Mean	8.36 ± 2.65	9.29 ± 3.00 *	10.41 ± 3.87 **,$
N	31	31	31

*: *p* < 0.05, **: *p* < 0.01 compared with before; $: *p* < 0.05 forest bathing vs. city walking.

**Table 13:** Impacts of forest and city walks on IGF-I in plasma (ng/mL) (Mean ± SD).

	Before (16 June)	After City Walking	After Forest Bathing
Mean	150.19 ± 46.92	149.52 ± 50.95	156.06 ± 55.07 *
N	31	31	31

*: *p* < 0.05 compared with city walking by paired *t*-test.

**Table 14:** Impacts of forest and city walks on SDS scores (Mean ± SD).

	Recruit	Before (16 June)	After City Walking	After Forest Bathing	After 1 Week
Mean	50.29 ± 6.09	48.61 ± 8.27	43.84 ± 9.64 **	40.74 ± 8.72 **,##	43.20 ± 12.16 **
N	31	31	31	31	31

**: *p* < 0.01 compared with before; ##: *p* < 0.01 forest bathing vs. city walking by paired *t*-test; “Recruit” means the SDS at the time of recruiting participants. “Before” means that the SDS was determined as before walking in the city area or forest bathing on 16 June. “After 1 week” means one week after the trip.

**Table 15:** Impact of forest and city walks on scores in POMS (Mean ± SD).

	AH	CB	DD	FI	TA	VA	F	TMD
Before 16 June	2.16 ± 3.62	5.55 ± 4.86	4.36 ± 5.06	7.71 ± 5.56	6.26 ± 5.05	5.74 ± 4.84	8.77 ± 4.32	20.42 ± 22.98
City walking	1.48 ± 3.15	4.35 * ± 4.8	3.58 ± 5.03	9.16 ± 4.67	4.16 ** ± 4.81	6.74 * ± 5.78	9.65 ± 5.40	16 ± 23.05
Forest bathing	0.74 **,$ ± 2.77	3.26 **,$ ± 4.53	2.87 * ± 4.31	4.61 **,$$ ± 4.21	2.94 **,$ ± 4.12	10.55 **,$$ ± 5.21	11.03 **,$$ ± 5.06	2.90 **,$$ ± 21.43

*: *p* < 0.05, **: *p* < 0.01 compared with before; $: *p* < 0.05, $$: *p* < 0.01 forest bathing vs. city walking (Mean + SE, n = 31). AH: anger–hostility, CB: confusion–bewilderment, DD: depression–dejection, FI: fatigue–inertia, TA: tension–anxiety, VA: vigor–activity, F: friendliness, TMD: total mood disturbance.

**Table 16:** Impacts of forest and city walks on subjective fatigue scores (Mean ± SD).

Groups	Before (16 June)	After City Walking	After Forest Bathing
Group 1	5.29 ± 2.04	5.19 ± 2.40	3.35 ± 2.29 **,$$
Group 2	4.10 ± 2.96	3.26 ± 3.51 *	2.71 ± 3.12 **
Group 3	2.55 ± 1.55	2.61 ± 1.60	1.58 ± 1.29 **
N	31	31	31

*: *p* < 0.05, **: *p* < 0.01 compared with before; $$: *p* < 0.01 forest bathing vs. city walk.

**Table 17:** Impacts of forest and city walks on sleep quality.

		Forest Bathing (Mean ± SD)	N	City Walking (Mean ± SD)	N
Factor I	Before	39.15 ± 6.83	16	38.98 ± 8.8	15
	After	45.77 ± 10.84 *	16	43.91 ± 10.88	15
Factor II	Before	39.03 ± 13.32	16	40.97 ± 11.8	15
	After	42.06 ± 11.77	16	40.68 ± 11.24	15
Factor III	Before	41.74 ± 16.21	16	48.11 ± 13.85	15
	After	43.26 ± 12.59	16	40.27 ± 14.83 *	15
Factor IV	Before	40.2 ± 10.08	16	42.34 ± 7.77	15
	After	45.79 ± 9.3	16	45.38 ± 11.69	15
Factor V	Before	41.15 ± 8.92	16	41.85 ± 15.75	15
	After	51.14 ± 12.49 *	16	51.25 ± 10.53	15

*: *p* < 0.05 compared with before by paired *t*-test. Factor I: Sleepiness on rising, Factor II: Initiation and maintenance of sleep, Factor III: Frequent dreaming, Factor IV: Feeling refreshed (recovery from fatigue), Factor V: Sleep length.

## Data Availability

Data in this study are available from the corresponding author upon request.
